# Complementary and conventional providers in cancer care: experience of communication with patients and steps to improve communication with other providers

**DOI:** 10.1186/s12906-017-1814-0

**Published:** 2017-06-08

**Authors:** Trine Stub, Sara A. Quandt, Thomas A. Arcury, Joanne C. Sandberg, Agnete E. Kristoffersen

**Affiliations:** 1 The National Research Center in Complementary and Alternative Medicine (NAFKAM), UiT The Arctic University of Norway, Department of Community Medicine, Tromsø, Norway; 2 Wake Forest School of Medicine, Department of Epidemiology and Prevention, Division of Public Health Sciences, Winston-Salem, NC 27157 USA; 3 Wake Forest School of Medicine, Department of Family and Community Medicine, Winston-Salem, NC 27157 USA

**Keywords:** Interdisciplinary communication, Complementary therapies, Conventional medicine, Cancer care

## Abstract

**Background:**

Effective interdisciplinary communication is important to achieve better quality in health care. The aims of this study were to compare conventional and complementary providers’ experience of communication about complementary therapies and conventional medicine with their cancer patients, and to investigate how they experience interdisciplinary communication and cooperation.

**Method:**

This study analyzed data from a self-administrated questionnaire. A total of 606 different health care providers, from four counties in Norway, completed the questionnaire. The survey was developed to describe aspects of the communication pattern among oncology doctors, nurses, family physicians and complementary therapists (acupuncturists, massage therapists and reflexologists/zone-therapists). Between-group differences were analyzed using chi-square, ANOVA and Fisher’s exact tests. Significance level was defined as *p* < 0.05 without adjustment for multiple comparisons.

**Result:**

Conventional providers and complementary therapists had different patterns of communication with their cancer patients regarding complementary therapies. While complementary therapists advised their patients to apply both complementary and conventional modalities, medical doctors were less supportive of their patients’ use of complementary therapies. Of conventional providers, nurses expressed more positive attitudes toward complementary therapies. Opportunities to improve communication between conventional and complementary providers were most strongly supported by complementary providers and nurses; medical doctors were less supportive of such attempts. A number of doctors showed lack of respect for complementary therapists, but asked for more research, guidelines for complementary modalities and training in conventional medicine for complementary therapists.

**Conclusion:**

For better quality of care, greater communication about complementary therapy use is needed between cancer patients and their conventional and complementary providers. In addition, more communication between conventional and complementary providers is needed. Nurses may have a crucial role in facilitating communication, as they are positive toward complementary therapies and they have more direct communication with patients about their treatment preferences.

## Background

Effective interdisciplinary communication between different groups of health care providers is essential to provide coordinated care [1, 2]. Optimal communication between health care providers is characterized by the accurate gathering and sharing of information. This includes what the patient has been told, planning who will take ongoing responsibility for the patient, and keeping the door open to further communication [3, 4].

Patient-centered communication is the set of skills and behaviors used by health care providers to promote relationships in which patients actively participate as partners in health care decision making and management [5, 6]. Recognition is growing throughout the medical and scientific research communities that an interdisciplinary approach to cancer prevention and control should incorporate patient-centered communication. This approach may maximize the benefit of current medical discoveries in diagnoses and treatment [2, 7].

Effective patient-provider communication may be essential in developing patient satisfaction, compliance, and positive health outcomes. Enhancing patients’ expectations through positive information about the treatment or illness, while providing support or reassurance, may influence health outcomes [8]. A recent Norwegian study among complementary therapy users who are cancer survivors suggests that effective communication may lead to the decision to use complementary therapy as a supplement rather than an alternative to conventional medicine [9]. In contrast, ineffective communication with conventional health care providers may result in the decision to use complementary therapy and even to delay or decline conventional cancer treatment.

A commonly described element of patient-provider communication is a *whole person* approach to patient care. The provider attends not only to the patient’s physical needs, but also to the psychological, social and behavioral dimensions of health and illness [10]. This type of communication plays a critical role in determining who will engage in health-enhancing lifestyles that reduce cancer risk [7]. Hence, a fruitful patient-provider relationship involves the concepts of mutuality, meaning, power sharing and collaboration between health care providers and patients.

Research reveals that only a small percentage of complementary therapists contact their patients’ physicians [11]. It is problematic that complementary therapists and conventional health care providers use different terminology when sharing information about patients. Poor physician-complementary therapist dialogue and limited patient disclosure of complementary therapy use create a *Bermuda Triangle* phenomenon, where important information may disappear [12]. This situation leaves it to the patients, who are in a vulnerable situation, to choose how to best integrate the two practitioner worlds [9].

A systematic review [8] investigating contextual effects of health in consultations between physicians and patients concluded that greater focus on research regarding human aspects is needed to achieve better quality in health care. To provide safe and coordinated care, we argue that interdisciplinary communication between different groups of health care providers is an important part of the contextual effects of health and therefore important to study. This is poorly investigated and needs to be explored through qualitative as well as quantitative approaches. Using data obtained through a survey, we describe aspects of the communication patterns between different groups of health care providers involving cancer patients’ use of complementary therapies. In this paper, our aims are to:

1. Compare conventional health care providers’ (oncology experts [doctors and nurses], family physicians) and complementary therapists’ (massage therapists, acupuncturists and reflexologists/zone therapists) experience of communication about complementary therapies and conventional medicine with their cancer patients.
2. Investigate how health care providers experience interdisciplinary communication and cooperation between complementary and conventional providers.

## Method

Norway residents receive healthcare within the public health care system, in which licensed conventional health care providers treat and care for patients [13]. Complementary therapies are practiced outside this system. Complementary practices are unregulated; anyone is allowed to use the term complementary therapist and treat patients [14]. However, most complementary therapists are members of a professional association. To ensure patient safety in cases of intervention-related health issues, the complementary therapists are required to obtain professional liability insurance. The patients generally cover the costs of visits to complementary therapists.

Data collection for this study included vanguard and main components. The Vanguard Study was completed in October 2015. It provided information on the questionnaire face and content validity [15] and informed the sampling procedures. The Main Study was completed from March to June 2016. The study protocol was reviewed and the Regional Committees for Medical and Health Research Ethics (REC) decided that the study did not need REC approval (2012/1318/REK Nord).

### Participants

Study inclusion criteria were being a currently practicing oncology doctor, oncology nurse, family physician, or complementary therapist. Complementary therapists could be an acupuncturist, massage therapist, or reflexologist/zone-therapist. Participants had to be members of a professional association. They had to have current or previous clinical experience with cancer patients.

#### Vanguard study

**Fig. 1:**
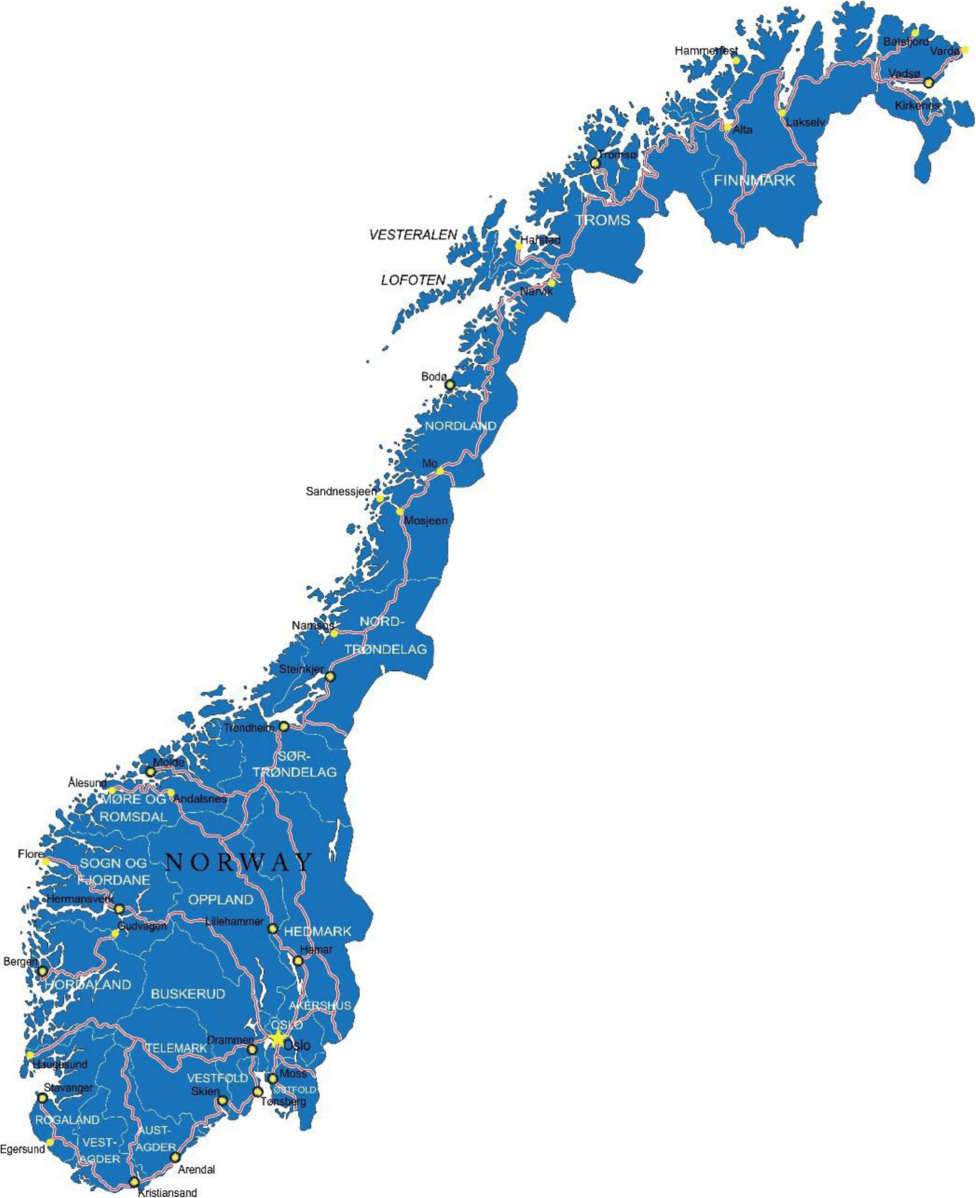
The study providers for the Vanguard Study were recruited from Troms County. For the Main Study, providers were recruited from Telemark and Vestfold Counties in the south, and Finnmark and Troms Counties in the north of Norway. This map is obtained from Colorbox. Colorbox is a digital image database, which the entire UiT The Arctic University of Norway, has been granted access to, free of charge. No copyright issue

**Fig. 2:**
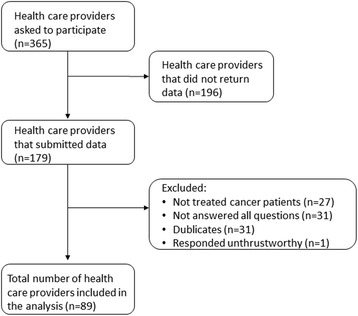
Flowchart of the inclusion process in the Vanguard Study

Oncology doctors and oncology nurses were recruited through the University Hospital of North Norway (UNN), located in Tromsø, a city in Troms County (Fig. 1). Family physicians were recruited through the Norwegian Medical Association, Troms County. Complementary therapists were recruited through the Norwegian Acupuncture Association (NAA), the Norwegian Association of Massage Therapists (NMF), and the Norwegian Association of Natural Medicine (NNH). The intended initial sample for the Vanguard Study included 106 study participants: 19 oncology doctors, 18 oncology nurses, 9 family physicians, 10 massage therapists, 20 acupuncturists, 10 hands on healers and 20 reflexologists/zone-therapists. However, due to an error, the survey was distributed to 289 reflexologists/zone-therapists instead of 20. Consequently, a total of 375 participants received an email and were asked to participate in the survey. A total of 179 participants completed and submitted the questionnaire, but 90 participants were excluded because they had not treated cancer patients (*n* = 27), had not completed all questions (*n* = 31), were duplicates (*n* = 31), or their responses were deemed untrustworthy (*n* = 1) (Fig. 2). The 89 Vanguard participants included 6 oncologists, 7 oncology nurses, 6 family physicians, and 70 complementary therapists (Table 1). It was not possible to differentiate complementary therapists into specific modalities, as individuals often indicated they practiced two or three modalities. Due to the inclusion criteria in the main study, we did not include the hands on healers in the main study.

**Table 1:** Sample design

	Vanguard study		Main study		Total	
	Number contacted	Number responded	Number contacted	Number responded	Number contacted	Number responded
Oncologists	19	6	130	26	149	32
Oncology nurses	18	7	125	111	143	118
Family physicians	9	6	454	118	463	124
Complementary therapists (includes acupuncturists, massage therapists, reflexologists/zone therapists)	329	70	632	262	961	332
Total	375	89	1332	517	1716	606

#### Main study

For the Main Study, oncologists and oncology nurses were recruited through the UNN in Troms County and the Vestfold Hospital Trust in Vestfold County. The counties of Troms and Vestfold have 164,330 and 244,967 inhabitants, respectively. Family physicians were recruited through the Norwegian Health Economics Administration (Helfo) Myfamilyphysician webpage (https://helfo.no). This public webpage lists the names and postal addresses of all physicians in Norway. We randomly selected two counties, Telemark and Finnmark, from the list and forwarded the questionnaire to family physicians practicing in these counties. Telemark and Finnmark counties have populations of 170,000 and 75,000 inhabitants, respectively. Complementary therapists were recruited using lists provided by the NAA, the NMF and the NNH.

A total of 1341 participants received an email or a letter with the questionnaire and were asked to participate in the survey. A total of 534 completed and submitted the questionnaire, but 17 participants were excluded because they were duplicates (*n* = 11) or did not indicate profession (*n* = 6).

**Fig. 3:**
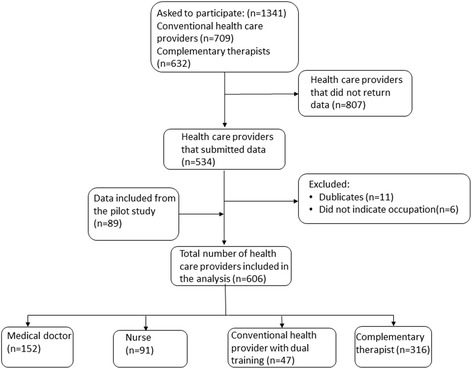
Flow chart of the inclusion process in the Main Study

The sample for the Main Study included 130 oncologists (26 responded), 125 oncology nurses (111 responded), 454 family physicians (118 responded), and 632 complementary therapists (279 responded) (Fig. 3). The final sample, including Vanguard and Main Study participants, consisted of 32 oncologists (21.5% response rate), 118 oncology nurses (82.5% response rate), 124 family physicians (26.8% response rate), and 279 complementary therapists (44.1% response rate). Participants could share the questionnaire link with others. Therefore, the actual denominator may be larger than the number of participants contacted, and the response rates would be correspondingly lower.

### Data collection

This study was a self-administrated questionnaire-based cross-sectional study.

#### Questionnaire content

The Vanguard Study questionnaire was based on information retrieved from a literature review performed by our research group [16]. During a 7 month research stay in the US, TS participated in weekly meetings with SQ, JS, and TA, where the questions were discussed and refined with input from a reference group of US conventional and complementary providers. In addition, a reference group in Norway (researchers and cancer patients) commented on the questions. The final version included 86 questions in 8 categories: inclusion (3 questions), communication with patients (20 questions), risk in clinical practice (26 questions), perceptions about complementary and conventional treatment modalities in cancer care (12 questions), information gathering about complementary and conventional therapies (6 questions), personal demographics (5 questions), clinical practice or hospital work (7 questions), and ethnicity (7 questions).

The Vanguard Study participants reported several overlapping questions and overly complicated response options. Consequently, we reduced the total number of questions from 86 to 65 for the Main Study. The Main Study questionnaire included 3 items for inclusion, 18 items on communication with patients, 14 items on risk in clinical practice, 12 items on perceptions about complementary and conventional treatment modalities in cancer care, 6 items on information gathering about complementary and conventional therapies, 5 items on personal demographics, and 7 items on clinical practice or hospital work. Thirteen questions were open ended, which allowed the participants to respond to the questions using their own words. We use a subset of the Main Study questions in this analysis.

#### Data collection procedures

Data collection for both the Vanguard and Main Studies was based on Dillman survey procedures [17]. In an electronic letter, the participants were invited to participate in the study. This letter informed the recipients that they would receive a request to help with an important study. One week later, e-mails were sent to all potential participants with a link to the online survey. After a week, an electronic “thank you” or reminder to complete the survey was sent to the selected providers using the same format that was used for the previous contact. Finally, one week later a reminder letter with a link to the survey was sent to the non-responders by e-mail. Following the same format, the physicians received the questionnaire by post, but with the option to complete the questionnaire either by post or email.

### Measures

Responders had to check at least one of the following options to be eligible: “Are you (check all that apply): oncology doctor; oncology nurse; family physician; physician; acupuncturist; massage therapist; reflexologist/zone-therapist.” In addition, they had to answer “Yes” to the two questions: “Do you ever treat patients who have been diagnosed with cancer?” and “Do you practice in Norway?” If they answered “No” to one of these questions, they were asked to complete only the questions about demographics, and they are not included in the data analyzed here. The question about treating patients diagnosed with cancer was intentionally broad to capture all practitioners interacting with cancer patients, whether or not that were providing treatment specific to the cancer.

Participants who checked oncology doctor or family physician but checked no other profession were classified as medical doctor in the analysis. Participants who checked oncology nurse or nurse but checked no other profession, were classified as nurse. Participants who checked any of the conventional health care categories and checked acupuncturist, massage therapist, or reflexologist/zone-therapy were classified as conventional health care provider with dual training. Participants who checked acupuncturist, massage therapist, or reflexologist/zone-therapist but checked no other profession were classified as complementary therapists.

Demographic information included age (years), sex (female, male) and level of education (both conventional and complementary training). Information about clinical practice included the number of patient and cancer patient visits in one week, practicing within a group or alone, working full time or part time, and the practice/hospital location (rural area, small city or large city).

Information about communication included four topics. Communication with patients was measured with 3 questions: Two questions that asked how often the provider asked patients if they used complementary therapies and how often they asked patients about what outcomes they expected from complementary therapies. Response options were never, sometimes, often and always. Often and always were combined for analysis. A third question asked what outcomes patients expected from complementary therapies, with dichotomous outcomes for: cure the cancer, slow progression of cancer, strengthen the immune system, symptom relief, reduce adverse effects of chemotherapy, improve physical and emotional wellbeing, provide hope and a sense of control. Participants could also respond, “I do not ask my patients about this.”

How to improve communication with patients was measured with 2 questions asking how the participant advised cancer patients if they asked about complementary therapies or about conventional treatment. Response options were to discourage use, encourage use, neither encourage nor discourage use, or to do something else.

Difficulty in communication was measured with 4 questions. Two items asked: how comfortable are you answering questions about complementary therapies? … about conventional cancer treatment? Two asked about difficulty with patients: How difficult do you find patients using complementary therapies? … using conventional medicine? All four questions used the response options not at all, moderately, and very.

Five options for steps that could improve communication between conventional and complementary providers were queried. Options were conventional training for complementary therapy providers, complementary therapy training for conventional providers, use of common terminology, including a complementary therapy provider in conventional practice, and including a conventional provider in complementary therapy practice. Responses were dichotomous (yes/no).

Four questions were open ended, allowing the participants to answer in their own words with options beyond those provided in the questionnaire: What else (1) would improve communication between complementary and conventional health care providers? (2) …do your cancer patients expect as outcomes from complementary therapy? How else (3) do you advise your cancer patients if they ask you about a complementary therapy? (4) …do you advise your cancer patients if they ask you about conventional treatment? Some of the responses were also used to illustrate and elaborate on the quantitative data.

### Statistical analyses

The analysis for this paper focuses on communication about complementary therapies and conventional medicine. Descriptive statistics (counts, percentages) were calculated, both overall and among the four practitioner groups. χ2 tests, Fisher exact test and logistic regression were used for analyzing binary dependent variables, and analysis of variance analyzed continuous, dependent variables. Significance level was defined as *p* < 0.05 without adjustment for multiple comparisons. The analysis was conducted using the SPSS V.19.0 for Windows. Respondents’ written comments and suggestions were analyzed using descriptive qualitative analysis [18].

## Results

### Demographics

Forty-five percent of the study participants were conventional health care providers (*n* = 274), including oncology doctors (*n* = 32), family physicians (*n* = 124) and nurses (*n* = 118). Half of the oncologists reported that they were male, and the majority of the family physicians, nurses, acupuncturists, massage therapists and reflexologists/zone therapists were female (Table 2).

**Table 2:** Sex according to profession

Profession	Total		Females		Males		No sex reported	
	n	(%)^a^	n	(%)	n	(%)	n	(%)
Oncology doctor	32	(5.3)	7	(21.9)	16	(50.0)	9	(28.1)
Family physician	124	(20.5)	63	(50.8)	56	(45.2)	5	(4.0)
Nurse	118	(19.5)	70	(59.3)	0	(0.0)	48	(40.7)
Conventional health care provider with dual training	1	(0.2)	1	(100)	0	(0.0)	0	(0.0)
Acupuncturist	212	(35.0)	126	(59.4)	30	(14.2)	56	(26.4)
Massage therapist	128	(21.1)	75	(58.6)	17	(13.3)	36	(28.1)
Reflexologist/zonetherapist	42	(6.9)	29	(69.0)	3	(7.1)	10	(23.8)

^a^Percentage of the total studied population reporting their profession. Due to the possibility of multiple answers, the sum exceeds 100%

Forty-seven individuals had dual training in conventional health care and complementary therapy (Table 3). More than half of the respondents (52%) were complementary therapists (*n* = 316). They were trained as acupuncturists (*n* = 212), massage therapists (*n* = 128) and reflexologists/zone-therapists (*n* = 42). The majority of the complementary therapy participants were trained in several modalities, so the reported numbers exceed the total number of participants in each provider group.

**Table 3:** Characteristics of the participants (*n* = 606)^a^

	Total		Medical doctor (*n* = 152)		Nurse (*n* = 91)		Conventional health care provider with dual training (*n* = 47)		Complementary therapist (*n* = 316)		*p*-value
	n	(%)	n	(%)	n	(%)	n	(%)	n	(%)	
Age, years											0.003^b^
Mean age	439	(48.2)	133	(45.5)	52	(50.4)	28	(50.7)	226	(48.9)	
Level of education											<0.0001^d^
Compulsory	2	(0.4)		(0.0)		(0.0)		(0.0)	2	(0.8)	
Middle level	45	(9.6)		(0.0)		(0.0)		(0.0)	45	(18.5)	
University up to 4 years	143	(30.4)		(0.0)	28	(50.0)	14	(45.2)	101	(41.6)	
University more than 4 years/PhD	280	(59.6)	140	(100)	28	(50.0)	17	(54.8)	95	(39.1)	
Profession^a^											
Oncology doctor	32	(16.2)	32	(100)							
Family physician	124	(59.6)	121	(97.6)			3	(2.4)			
Nurse	118	(41.5)			91	(100)	27	(93.1)			
Conventional health care provider with dual training	1	(0.6)					1	(100)			
Acupuncturist	212	(57.5)		(0.0)		(0.0)	38	(17.9)	174	(82.0)	
Massage therapist	128	(46.7)		(0.0)		(0.0)	9	(75.0)	119	(73.0)	
Reflexologist/zonetherapist	42	(22.5)		(80.0)		(0.0)	3	(7.1)	36	(92.8)	
Clinical practice											<0.0001^d^
Full time health provider	287	(71.9)	121	(89.0)	38	(77.6)	18	(72.0)	110	(58.2)	
Part time health provider	92	(23.1)	11	(8.1)	10	(20.4)	5	(20.0)	66	(34.9)	
Other (students or retired persons)	20	(5.0)	4	(2.9)	1	(2.0)	2	(8.0)	13	(6.9)	
Patient visits per week											<0.0001^c^
1–19 patients	132	(33.9)	11	(8.3)	27	(57.4)	4	(16.0)	90	(48.6)	
20–39 patients	121	(31.1)	28	(21.2)	17	(36.2)	5	(20.0)	71	(38.4)	
40 or more patients	136	(35.0)	93	(70.5)	3	(6.4)	16	(64.0)	24	(13.0)	
Cancer patient visits per week											<0.0001^d^
1–19 cancer patients	362	(92.1)	126	(92.6)	31	(64.6)	23	(92.0)	182	(98.9)	
20 and more patients	31	(7.9)	10	(7.4)	17	(35.4)	2	(8.0)	2	(1.1)	
Location											0.004^c^
Rural area	119	(29.9)	57	(41.9)	7	(14.6)	3	(12.0)	52	(27.5)	
Small city, village (up to 50,000 inhabitants)	153	(38.4)	44	(32.4)	23	(47.9)	12	(48.0)	74	(39.2)	
Large city (>50,000 inhabitants)	126	31.7)	35	(25.7)	18	(37.5)	10	(40.0)	63	(33.3)	

^a^Due to multiple response on one or more variables, the analyzed numbers do not always add up to the total number

^b^One-way ANOVA

^c^Chi-square

^d^Fisher’s exact test

A significant difference was found between the provider groups in age, with medical doctors and complementary therapists being, on average, younger than the other groups (*p* = 0.003). A significant difference was also found between the groups regarding education. More than four years of education was reported by 100% of the medical doctors, 55% of the primary health care providers with dual training, and 50% of the nurses, compared to only 39% of the complementary therapists (*p* < 0.0001).

The majority of the medical doctors and nurses worked full time (89% and 78%, respectively). These numbers were significantly greater than those for the complementary therapists; 58% worked full time, while 35% worked part time (*p* < 0.0001).

Seventy percent of the medical doctors and 64% of the conventional health care providers with dual training had 40 or more patient visits per week, compared to only 13% of the complementary therapists and 6% of the nurses (*p* < 0.0001). More than 90% of the medical doctors, providers with dual training and the complementary therapists had between 1 and 19 cancer patient visits per week; by comparison, 35% of the nurses had 20 or more cancer patient visits (*p* < 0.0001).

The largest proportion of medical doctors (42%) practiced in rural areas. The largest proportion of nurses (48%), providers with dual training (48%) and complementary therapists (39%) worked in small towns (*p* = 0.004).

### Communication with cancer patients about treatments

Overall, the communication of health care providers with their cancer patients about treatment modalities followed provider specialty. Responses to the questions were frequently distributed in two opposite ends of the scales, with conventional providers on one end and the providers with dual training and the complementary therapists on the other end (Table 4).

**Table 4:** Communication with patients (*n* = 606)^a^

	Total		Medical doctor (*n* = 152)		Nurse (*n* = 91)		Conventional health care provider with dual training (*n* = 47)		Complementary therapist (*n* = 316)		*p*-value
	n	(%)	n	(%)	n	(%)	n	(%)	n	(%)	
How often do you ask your cancer patients it they use CT?											.000^c^
Never	53	(13.3)	29	(21.0)	9	(15.8)	1	(4.0)	14	(7.8)	
Sometimes	152	(38.0)	84	(60.9)	33	(57.9)	6	(24.0)	29	(16.1)	
Often/always	195	(48.8)	25	(18.1)	15	(26.4)	18	(72.0)	137	(76.1)	
How often do you ask your patients about what outcome they expect from CT?											.000^c^
Never	36	(8.9)	23	(16.9)	4	(7.1)	1	(3.8)	8	(4.3)	
Sometimes	148	(36.5)	71	(52.2)	34	(60.7)	8	(30.8)	35	(18.7)	
Often/Always	221	(54.6)	42	(30.9)	18	(32.2)	17	(65.3)	144	(77.0)	
Do your cancer patients expect the following outcomes from CT?											
cure the cancer	131	(21.7)	86	(57.0)	35	(38.5)	1	(2.1)	9	(2.8)	.000^b^
slow the progression of cancer	174	(28.8)	92	(60.9)	40	(44,0)	3	(6.4)	39	(12.3)	.000^b^
strengthen the immune system	243	(40.2)	71	(47.0)	37	(40.7)	19	(40.4)	116	(37.7)	.209^b^
symptoms relief	274	(45.3)	80	(53.0)	32	(35.2)	22	(46.8)	140	(44.3)	.057^b^
reduce adverse effects of chemo therapy	236	(39.0)	58	(38.4)	27	(29.7)	22	(46.8)	129	(40.8)	.172^b^
improve physical and emotional well being	272	(45.0)	65	(43.0)	28	(30.8)	22	(46.8)	157	(49.7)	.014^b^
provide hope and a sense of control	171	(28.3)	59	(39.1)	29	(31.9)	11	(23.4)	72	(22.8)	.002^b^
I do not ask my patients about this	23	(3.8)	13	(8.6)	3	(3.3)	0	(0.0)	7	(2.2)	.006^c^
How do you advice your cancer patients if they ask you about CT?											.000^c^
Discourage use	14	(3.6)	10	(7.4)	2	(3.6)	0	(0.0)	2	(1.1)	
Encourage use	100	(25.6)	3	(2.2)	4	(7.3)	11	(42.3)	82	(47.1)	
Neither	177	(45.4)	81	(60.0)	30	(54.5)	10	(38.5)	56	(32.2)	
Other	99	(25.4)	41	(30.3)	19	(34.5)	5	(19.2)	34	(19.5)	
How do you advise your cancer patients if they ask you about conventional treatment?											.002^c^
Discourage use	2	(0.5)	0	(0.0)	0	(0.0)	0	(0.0)	2	(1.1)	
Encourage use	309	(78.2)	117	(86.7)	42	(77.8)	21	(84.0)	129	(71.3)	
Neither	46	(11.6)	5	(3.7)	6	(11.1)	4	(16.0)	31	(17.1)	
Other	38	(9.6)	13	(9.6)	6	(11.1)	0	(0.0)	19	(10.5)	

^a^Due to multiple response to some of the questions and lack of response from participants the analyzed numbers do not always add up to the total number

^b^Pearson Chi-square test

^c^Fisher’s exact test

Only 18% of the medical doctors and 26% of the nurses asked their cancer patients *often/always* if they used complementary therapies, while 76% of the complementary providers and 72% of the providers with dual training asked *often/always* (*p* < 0.0001) (Table 4). Thirty-one percent of the medical doctors and 32% of the nurses *often/always* asked their patients about what outcome they expected from complementary therapies. Significantly more of the providers with dual training (65%) and the complementary therapists (77%) *often/always* asked about this (*p* < 0.0001).

The contrast was striking between conventional providers and complementary therapists in the outcomes they believed their patients expected from complementary therapy. The majority of medical doctors believed that curing the cancer (57%) and slowing the progression of cancer (61%) were outcomes patients expected from complementary therapies, while, respectively, less than 3% and 7% of providers with dual training and 3% and 13% of complementary therapists believed that (*p* < 0.0001). Fewer between-group differences were found in patients’ expecting a stronger immune system, where less than half of each group thought patients expected this, and symptom relief, where between a third and a half of providers believed patients expected this. Similarly, about 40% of each group thought reducing the adverse effects of chemo therapy was expected by patients using complementary therapies. More complementary therapists and providers with dual training reported that improved physical and emotional wellbeing was an outcome expected by patients using complementary therapies than did providers with conventional training (*p* = 0.014). In contrast, more conventional providers (39% of medical doctors and 32% of nurses) thought patients using complementary therapies expected these therapies to provide hope and a sense of control, compared with only 23% of dual trained providers and complementary therapists (*p* = 0.002).

A total of 157 participants responded to an open-ended question about what else might improve communications between conventional health care providers and complementary therapists. Of these were (*n* = 27) medical doctors, (*n* = 8) nurses, (*n* = 17) providers with dual training and (*n* = 106) complementary therapists. One complementary therapist added:

*Occasionally strengthening the immune system, but it depends on whether the aim is to strengthen the patient’s immune system. This must be discussed with the oncologist. All patients [visiting my clinic] are informed that acupuncture can NOT heal the cancer itself (CT therapist).*

A medical doctor added:

*I don’t have cancer patients who use complementary therapies (MD).*

If patients asked for advice about complementary therapies, most medical doctors and nurses neither encouraged nor discouraged its use (60% and 54%, respectively) (Table 4). In contrast, when asked by patients about complementary therapy use, providers with dual training and complementary therapists tended to encourage their patients to use complementary therapies (42% and 47%, respectively (*p* < 0.0001). Seventy-eight percent of *all* participants encouraged their patients to use conventional cancer treatment when asked, though dual providers and complementary therapists were somewhat more likely to neither encourage nor discourage use (*p* = 0.002).

#### Difficulties in provider-patient communication

Only 16% of the medical doctors and 18% of the nurses were *very* comfortable answering questions about complementary therapies, compared to 60% of the providers with dual training and 71% of the complementary therapists (*p* < 0.0001) (Table 5). The majority of the medical doctors, nurses and providers with dual training were *very* comfortable answering their patients’ questions about conventional cancer treatment (55%, 70% and 50%, respectively), compared to only 34% of the complementary providers (*p* < 0.0001).

**Table 5:** Difficulties in provider-patient communication (*n* = 606)^a^

	Total		Medical doctor (*n* = 152)		Nurse (*n* = 91)		Conventional health care provider with dual training (*n* = 47)		Complementary therapists (*n* = 316)		*p*-value
	n	(%)	n	(%)	n	(%)	n	(%)	n	(%)	
How comfortable are you to answer question about CT?											.000^b^
Not at all	32	(6.9)	16	(11.3)	8	(11.8)	1	(3.3)	7	(3.2)	
Moderately	219	(47.4)	103	(72.5)	48	(70.6)	11	(36.7)	57	(25.7)	
Very	211	(45.7)	23	(16.2)	12	(17.6)	18	(60.0)	158	(71.2)	
How comfortable are you to answer question about conventional cancer treatment?											.000^b^
Not at all	34	(7.4)	4	(2.8)	1	(1.4)	1	(3.3)	28	(12.7)	
Moderately	210	(45.5)	60	(42.0)	20	(29.0)	14	(46.7)	116	(52.7)	
Very	218	(47.2)	79	(55.2)	48	(69.6)	15	(50.0)	76	(34.5)	
How difficult do you find patients using CT?											.000^b^
Not at all	250	(63.3)	52	(38.5)	33	(57.9)	20	(80.0)	145	(81.5)	
Moderately	137	(34.7)	81	(60.0)	24	(42.1)	4	(16.0)	28	(15.7)	
Very	8	(2.0)	2	(1.5)	0	(0.0)	1	(4.0)	5	(2.8)	
How difficult do you find patients using conventional medicine?											.000^b^
Not at all	318	(79.1)	83	(62.9)	44	(78.6)	21	(84.0)	170	(91.9)	
Moderately	75	(18.8)	48	(36.4)	12	(21.4)	3	(12.0)	12	(6.5)	
Very	5	(1.3)	1	(0.8)	0	(0.0)	1	(4.0)	3	(1.6)	

^a^Due to missing responses the sum does not always add up to the total number of 606

^b^Fisher’s exact test

Thirty-nine percent of the medical doctors found patients’ use of complementary therapies *not at all* difficult, compared to 58% of the nurses. More than 80% of the providers with dual training and complementary therapists found this *not at all difficult*.

Medical doctors (*n* = 41), nurses (*n* = 19), providers with dual training (*n* = 5) and complementary therapists (*n* = 34) wrote additional comments. Several of the medical doctors expressed that it depended entirely on the modality their patients wanted to use. One added:

*My advice depends on my knowledge and experience with the modality of my patient’s choice* (MD).

Moreover:

*I recommend conventional treatment, and if they want to use complementary therapies in addition, they should discuss this with the specialist health service* (MD).

Several added comments like this:

*I advise them to consider the cost-benefit and adverse effects carefully* (MD).

Some nurses (*n* = 6) and complementary therapists (*n* = 7) wrote that they advised the patients to talk with their conventional health care provider. For example, one said:

*I strongly recommend that they consult their oncologists and follow their advice!* (Nurse).

Several medical doctors had a positive attitude toward patient use of complementary modalities, but they qualified their advice with negative connotations. They stated:

*If harmless, I support their choice* (MD).

*If they don’t get fleeced for money and feel that it helps, it’s OK* (MD).

*I’m open to anything that works, and, as long as it’s not outrageous, go ahead!* (MD).

The nurses and providers with dual training supported the patients regardless of their choice:

*I listen to them, explain the effect they experience and take it further from there* (Physician with dual training).

Moreover, several wrote:

*I support my patients whatever they choose* (Nurse).

While the majority of providers in each group found their patients use of conventional medicine *not at all* difficult, slightly fewer of the medical doctors (63%) and nurses (79%) reported this acceptance than did providers with dual training (84%) and complementary therapists (92%) (*p* < 0.0001). Medical doctors (*n* = 30), nurses (*n* = 12) and complementary therapists (*n* = 36) wrote additional comments that illuminated the quantitative responses. Medical doctors (*n* = 17) discussed the pros and cons of conventional cancer treatment with their patients. They wrote statements like:

*It depends entirely on the cancer type. If the cancer isn’t curable, I often recommend forgoing conventional therapy, as adverse effects are often worse than living with the disease* (MD).

A nurse added:

*I inform and explain about conventional cancer treatment, why it is recommended and support the choice they make* (Nurse).

Some complementary therapists responded:

*I recommend that they talk to at least two different oncologists, ask about the effects and adverse effects and what they can expect from the treatment and recommend that they follow the oncologists’ advice* (CT).

*I don’t give advice regarding conventional cancer treatment, but tell them to talk with their family physician* (CT).

#### Actions that would improve communication between conventional and complementary health care providers

Respondents were queried about five different actions that could improve communications among health care providers. In general, complementary therapists and dual trained providers were the most supportive of these, with nurses and medical doctors less enthusiastic about the actions. Offering complementary providers training in conventional methods as a way to improve communication was supported by a greater proportion of providers with dual training (94%) and complementary therapists (93%) than conventional providers (medical doctors 68%, nurses 88%) (*p* < 0.0001) (Table 6). Similarly, providers with dual training and complementary therapists showed greater support for providing complementary therapy training to conventional providers than did medical doctors and nurses (*p* < 0.0001). Similar results were found for the use of common medical terminology (*p* < 0.0001). While only about half of the medical doctors (55%) supported including complementary therapists in conventional practice and vice versa, the majority of the nurses (85%), providers with dual training (100%) and complementary therapists (99%) supported this (*p* < 0.0001). When we asked what else could improve communication between conventional and complementary providers, several medical doctors added:

**Table 6:** Actions that would improve communication between conventional and complementary health care providers (*n* = 606)^a^

	Total		Medical doctor (*n* = 152)		Nurse (*n* = 91)		Conventional health care provider with dual training (*n* = 47)		Complementary therapist (*n* = 316)		*p*-value
	n	(%)	n	(%)	n	(%)	n	(%)	n	%	
Conventional training for CT providers											.000^c^
Yes	380	(84.6)	95	(68.3)	56	(87.5)	29	(93.5)	200	(93.0)	
No	69	(15.4)	44	(31.7)	8	(12.5)	2	(6.5)	15	(7.0)	
CT training for conventional providers											.000^c^
Yes	387	(86.2)	97	(69.8)	56	(84.8)	31	(100)	203	(95.3)	
No	62	(13.8)	42	(30.2)	10	(15.2)	0	(0.0)	10	(4.7)	
Use of common medical terminology											.000^b^
Yes	365	(81.3)	93	(67.4)	52	(78.8)	27	(87.1)	193	(90.2)	
No	84	(18.7)	45	(32.6)	14	(21.2)	4	(12.9)	21	(9.8)	
Including a CT provider in conventional practice											.000^c^
Yes	375	(83.3)	75	(54.7)	57	(85.1)	31	(100)	212	(98.6)	
No	75	(16.7)	62	(45.3)	10	(14.9)	0	(0.0)	3	(1.4)	
Including a conventional provider in CT practice											.000^c^
Yes	360	(80.7)	76	(55.5)	50	(76.9)	29	(96.7)	205	(95.8)	
No	86	(19.3)	61	(44.5)	15	(23.1)	1	(3.3)	9	(4.2)	

^a^Due to missing responses the sum does not always add up to the total number of 606

^b^Pearson chi-square test

^c^Fisher’s exact test

*Acceptance, respect and more research* (MD).

*Complementary therapists must have an understanding of the cause of disease that is compatible with “conventional medicine”*(MD).

*Complementary therapists must also realize their limitations* (MD).

*Evidence-based research/guidelines on complementary therapies* (nurse and MD).

One of them disagreed:

*Cannot see the need. We have different perspectives of the world (MD).*

Several encouraged the providers to be more tolerant:

*Listen to and hold each other accountable* (MD).

*Improve knowledge about each other’s fields. Lower the brick wall and ease the war atmosphere* (MD).

When we asked for other steps that may improve communication between different health care providers, the participants added that education and cooperation may improve communication.

*Acupuncturists with bachelor’s degrees can communicate better with other health personnel due to training in Western medicine* (CT).

*Mutual workshops, networks and seminars* (provider with dual training).

*Mutual assessments of the patients’ own experience with complementary therapies* (MD and nurse).

*Fruitful dialogue and good information both ways* (providers with dual training).

## Discussion

This study demonstrated consistent differences in how conventional and complementary providers experience communication about complementary therapies and conventional medicine with their cancer patients. Conventional providers were less likely than complementary therapists to ask their cancer patients about their use of and expected outcomes for complementary therapies. Conventional and complementary providers differed in what they thought their cancer patients using complementary therapies expected from the therapies, with conventional providers (but not complementary providers) assuming their patients expected cancer cure and reduced progression of the cancer through the use of complementary therapies. Complementary therapists were more likely than conventional providers to think their patients expected improved physical and emotional well-being from complementary therapies. As might be expected, complementary therapists were more likely than conventional providers to encourage their patients to use complementary therapists. However, these complementary therapists also recommended conventional treatments to their patients.

According to Ben-Arye et al., cancer patients should be asked about their complementary therapy use [19] because it will strengthen the patient-provider relationship by which therapeutic goals are achieved [6]. Moreover, patients favor health care providers with dual training [20, 21].

Medical doctors appeared to vary in their attitudes toward complementary therapies. Some claimed not to have patients who used complementary therapies; others had very positive attitudes towards these modalities. Many expressed (in the open-ended questions) that their advice depended on what modality the patients wanted to use, while nurses supported their patients whatever modality they wanted to apply. Risber et al. [22] found in a study from 2004 that more than half of the physicians (56%) and most of the other health care workers (85–93%) had a positive attitude toward departments of integrative medicine. However, major differences among the professionals were found.

A literature review by Stub et al. [16] reported that physicians will only discuss complementary therapies when a patient raises the issue within a consultation due to lack of scientific evidence derived from high quality Randomized Controlled Trials (RCT). They also endorse complementary modalities that are closest to conventional medicine [23], which is in line with our findings. Our findings show that providers are consistent with patient research that reported the following reasons for the use of complementary therapies in cancer care: Reduce pain related symptoms and adverse effects, strengthen the immune system or increase in the body’s ability to fight the disease [24], improve sleep, physical and/or emotional well-being and improvement of the quality of life [25].

The majority of the providers in this study encouraged their patients to use conventional cancer treatment. However, some of the medical doctors were concerned about continuing treatments that were directly cancer related when the cancer was incurable and the treatment was associated with substantial adverse effects. Factors that have been shown to contribute to patients’ lack of acceptance of medical facts include the lack of trust in physicians, the oncologist’s use of ambiguous language and alternative belief systems [26, 27]. Medical doctors may improve the patients’ understanding, but this may affect the patients’ satisfaction with them.

When we asked about steps that could improve communication between different types of providers, the majority of the providers responded positively to recommendations including more cross-training, common medical terminology and including a complementary therapist in a conventional practice and vice versa. King et al. reported in a survey from Canada that 80% of conventional health care providers expressed interest in receiving more training about complementary therapies [28]. Moreover, Klimenko et al. suggest that common medical terminology is a step to bridge the gap between conventional and complementary providers [29]. While a majority of providers in each group in our study thought that the identified steps could improve communication between different types of providers, medical doctors were notably less confident that the step would improve communication than the other provider groups. Further exploration of this finding is necessary.

In this study the nurses were open to complementary therapies. They were more likely than medical doctors to ask cancer patients about use of complementary therapies and their expected outcomes and they had greater expectation that steps would improve communication between different healthcare providers. This is in line with Richardson [30] who found that cancer nurses sometimes actively promote complementary therapies that they find to correspond with their vision of holistic care. According to Broom et al. [31], they are the patients’ advocate and they often mediate between oncologists and patients [30].

The qualitative data revealed a perceived need for more respect, cooperation between peers, and a need for mutual arenas to meet and communicate. Conventional providers called for more research and guidelines for complementary modalities, and training in pathology for complementary therapists. Barrett et al. [32] found that conventional and complementary providers often differ in their understanding of treatment concepts, philosophies and diagnostic procedures leading to different models of disease causality and treatment philosophy. This is in line with our findings. Even though, a number of the medical doctors in our study showed a lack of respect for complementary therapies.

### Practical implications

For the well-being of cancer patients, it is important to encourage provider-patient communication about the use of all types of treatments, both conventional and complementary. Similarly, cancer patients will be best served by open communication among all providers from whom they receive treatment. Reducing the communication gap can be facilitated by promoting greater communication of patients with their providers. Most likely, this needs to be encouraged by providers. The communication gap between different health care providers in cancer care can be reduced by creating common arenas where the providers can meet and communicate. This could be in shared practices or in joint educational experiences. Nurses could play a role in facilitating communication, both between patients and providers, and between different types of providers, as they are more sympathetic to complementary therapies and may have more direct communication with patients about their treatment preferences and use of complementary therapy.

### Limitations

The results of this analysis should be interpreted in light of the study’s limitations. The response rate was low, which may be a threat to the generalizability of the findings, because the non-responders may differ significantly from those who responded [33]. However, the findings about communication patterns between different health care providers are in line with other studies [23, 30, 31], which suggest that the nonresponse bias probably imposes no major threat to the validity of the results [34].

## Conclusion

Most medical doctors and nurses showed respect and acceptance towards cancer patients’ use of complementary therapies. Cross training, common medical terminology and inclusion of complementary therapists in conventional practice and vice versa, are steps that may enhance communication between conventional health care providers and complementary therapists. However, different worldviews and lack of acceptable evidence for complementary modalities may be a hindrance for fruitful communication between complementary therapists and conventional health care providers. Nurses may have a specific role in facilitating communication, as they are positive to complementary therapies and they have more direct communication with patients about their treatment preferences.
